# A Human-Centered Framework to Capture the Holistic Pain Story in Chronic Pain: Protocol for a Multicenter Observational Feasibility Study

**DOI:** 10.2196/95222

**Published:** 2026-10-09

**Authors:** Jodi Townsley Dubuclet, Krishna Badhiwala, David Page, Scott DeBates, Vivek Kumar Tyagi, Binesh Balasingh, Eric Carpenter, Kiran V Patel

**Affiliations:** 1 Research and Development, Neuromodulation, Abbott, 6901 Preston Rd., Plano, TX, 75024, United States, 1 2148434997, 1 9723098000; 2 Northwell Health, New York, NY, United States

**Keywords:** digital health, human-centered design, chronic pain, patient-physician communication, feasibility studies, artificial intelligence, AI, neuromodulation, qualitative research, digital biomarkers

## Abstract

**Background:**

Standardized metrics such as the numeric rating scale (NRS) provide essential quantitative pain benchmarks but can be augmented by capturing the functional and emotional context of a patient’s lived experience. Adding surveys creates a “contextual disconnect” (increasing data yield with diminishing insights) and adds patient burden. The CONNECT (Collecting Communication Data to Enhance Patient-Physician Interaction) study proposes a structured, multimodal framework integrating patient narratives, drawings, and mobility assessments to capture each patient’s holistic “pain story” and create a comprehensive ground truth dataset for developing digital health tools.

**Objective:**

This study aims to (1) evaluate the feasibility of capturing multimodal communication data (narratives, drawings, and mobility) from patients with chronic pain, (2) quantify gaps between standard clinical metrics (eg, the NRS) and patient-defined key functional limitations, and (3) evaluate the usability and perceived communication value of software prototypes (myStory).

**Methods:**

CONNECT is a multiphase, multicenter, exploratory feasibility study using human-centered design. Up to 3 tertiary care interventional pain practices in the United States will enroll 2 mutually exclusive cohorts (up to 40 patients each). Phase 1 conceptualizes the patient narrative as a “clinical sensor,” collecting a foundational ground truth dataset via video-recorded pain narratives, guided drawings, standardized mobility assessments, and validated surveys. Phase 2 uses phase 1 learnings to develop and evaluate up to 5 myStory prototypes, assessing utility, usability, and perceived communication value via task-based evaluation and think-aloud protocols. Exploratory analyses include dual-coder thematic analysis, pose estimation mobility assessments, and AI-assisted narrative summarization.

**Results:**

Recruitment began in May 2026. As of September 2026, one site is active, with 37 participants enrolled. Anticipated outcomes include (1) a rich, multimodal dataset establishing a ground truth of patient-derived functional limitations, goals, and expectations; (2) characterization of pain story gaps missed by the NRS via the contextual disconnect rate (defined as NRS ≤6 with ≥1 key functional limitation); (3) evaluation of at least one feasible prototype concept (mean score of 3.0 on a 5-point Likert scale); and (4) initial user testing of new communication assessment scales. Results are anticipated in late 2026 and will be disseminated through peer-reviewed publications and conferences.

**Conclusions:**

This protocol establishes a reproducible framework for understanding the holistic “pain story” by capturing narrative, visual, and functional dimensions of chronic pain. Findings will inform new communication tools intended to enhance patient-provider communication and align care with outcomes that matter most to patients with chronic pain.

## Introduction

From our preprotocol needs-gathering interviews with six patients with chronic pain, one patient with chronic pain stated the following:

It’s hard to explain. My pain score might be a 4, but I can’t stand long enough to cook a meal for my family. That’s what really matters.

This statement highlights a fundamental paradox in chronic pain management: the very tools used to measure a patient’s experience often fail to capture what matters most to them.

Clinical practice and research have long relied on the numeric rating scale (NRS) or visual analog scale to distill the complex, multidimensional human experience of pain into a single, easily documented statistic. While these instruments are convenient and widely adopted, they often miss the crucial context of a patient’s functional goals, emotional realities, and quality of life [1], and titrating treatments to maximize these survey outcomes alone can create an illusion of impact while a patient’s core needs may remain unaddressed. This disconnect can lead to patient frustration, suboptimal therapeutic alignment and health care waste, and unmet needs [2,3]. Furthermore, providers underscore persistent gaps in current outcome assessment instruments, including functional assessment limitations, limited context, and communication barriers, all domains this study is specifically designed to address [4].

In response to this, many recent research efforts have focused on a proliferation of digital health technologies to augment clinical surveys. This has created a “digital paradox”: an abundance of data without more meaningful insights. More data do not necessarily translate into better communication or improved outcomes. While text-only AI is feasible for patient intake [5,6] or identifying linguistic markers of pain from narratives that uncover dimensions invisible to standard scales [7,8], chronic pain requires more context. Patients’ pain narratives themselves are often incomplete: individuals selectively reveal or conceal certain aspects of their condition, and cultural and social filters shape what they choose to share, leaving key emotional and functional dimensions often unspoken. Together, this reinforces that chronic pain narratives provide important insights but are not sufficient on their own [9]. Our novel multimodal approach of analyzing how patients speak and move, not just what they say, is designed to capture this richer picture. Without this context, technology risks amplifying the “contextual disconnect” between what patients express and what clinicians perceive.

To address this challenge, we have designed the CONNECT (Collecting Communication Data to Enhance Patient-Physician Interaction) study. This study proposes that a patient’s true “pain story” is not a single number but a narrative of functional limitation, emotional consequences, and life goals. To capture this complexity, the CONNECT study introduces a computational multimodal framework (Figure 1) to capture ground truth data directly from patients by integrating three key data streams designed to capture verbal and nonverbal communication cues often lost in standard documentation [10]: video-recorded function-based pain narratives that capture verbal and nonverbal cues, video-recorded pain drawing exercises that allow patients to visually articulate their pain [11-15], and standardized mobility assessments that provide objective physical context.

**Figure 1.**
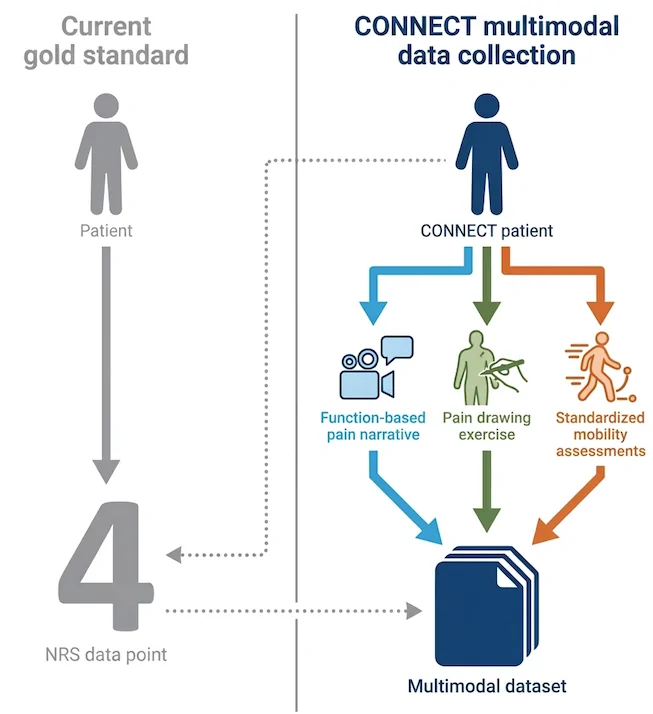
The CONNECT (Collecting Communication Data to Enhance Patient-Physician Interaction) framework—from pain score to holistic story. NRS: numeric rating scale.

This protocol describes the design of a multiphase study to evaluate the technical feasibility and early acceptability of a human-centered framework for chronic pain communication. The study has the following core aims:

- To assess the feasibility of collecting rich, multimodal communication data from patients with chronic pain
- To quantify any gaps between patient-reported pain intensity and functional limitations derived from phase 1 findings
- To evaluate the feasibility, utility, and perceived communication value of an elemental software prototype (myStory) derived from the phase 1 findings (phase 2)
- To propose new communication scales and perform an initial evaluation of them

## Methods

### Study Design

CONNECT is a multiphase, multisite, human observational study using a human-centered design process [16] that conceptualizes the patient’s unstructured narrative as a “clinical sensor” of their chronic pain experience. The study is structured into 2 distinct phases with separate, mutually exclusive cohorts of participants (n=40 per phase). This protocol is reported in accordance with the SPIRIT (Standard Protocol Items: Recommendations for Interventional Trials) 2025 statement. Participants will complete a single study visit with randomized ordering of a multimodal data collection session and a standard-of-care clinical visit (phase 1) or interactive prototype evaluation (phase 2), as detailed in Figure 2. Randomization sequence generation will be executed via a computer-generated block randomization schedule, and allocation concealment will be implemented using a secure, centralized electronic data capture system accessed by the research coordinator at the time of enrollment. A planned analysis of order effects will evaluate whether visiting the physician prior to the data collection session influenced patient responses compared to collecting data first.

**Figure 2.**
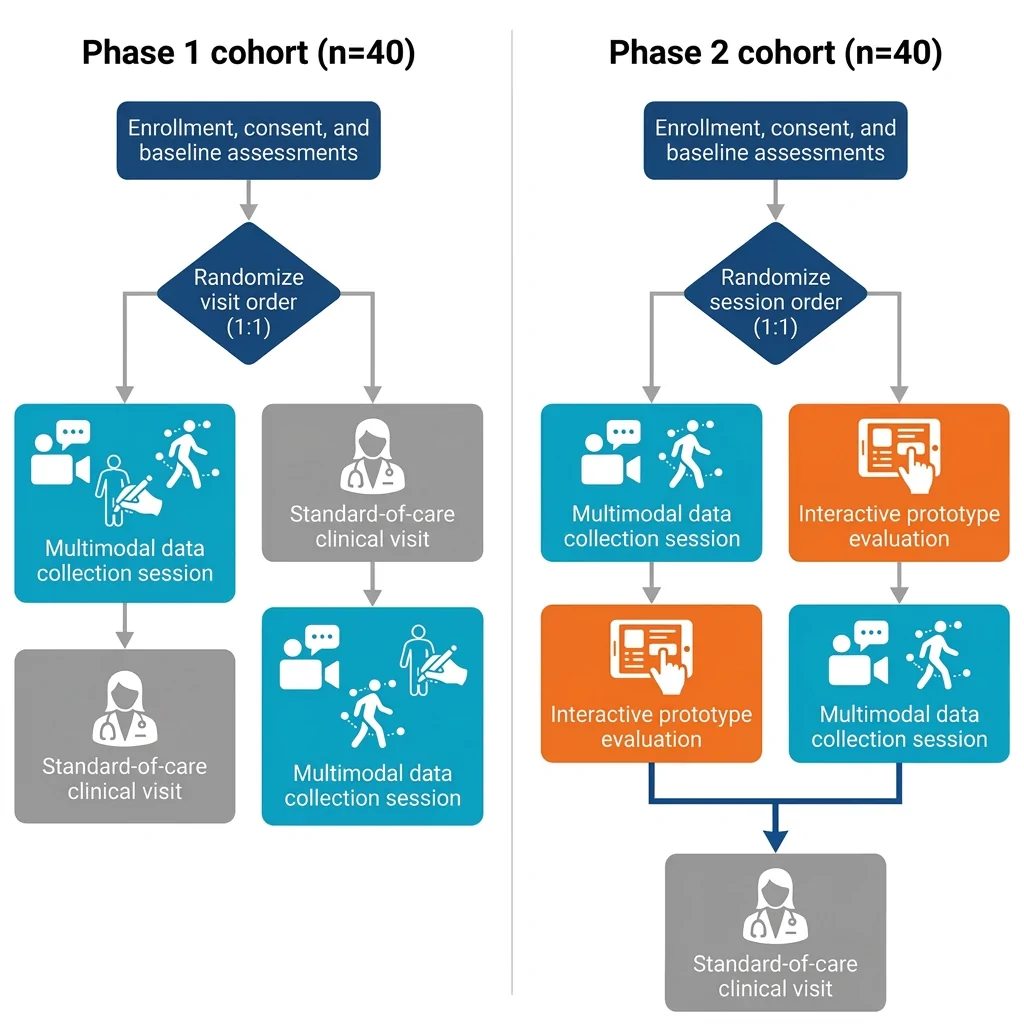
CONNECT (Collecting Communication Data to Enhance Patient-Physician Interaction) study patient flow for phase 1 (need and gap collection) and phase 2 (human factors study and prototype development).

To ensure participant safety during standardized mobility assessments (timed walk and sit-to-stand transitions), continuous safety monitoring will be conducted. Fall risk screening will be conducted by the investigator prior to enrollment; patients deemed at elevated risk of falling will be excluded. Participants are permitted to use their standard assistive devices (eg, canes and walkers) during testing. Specific stopping criteria include the participant requesting to stop, visible signs of severe pain exacerbation, or signs of emotional distress, at which point the research coordinator will immediately halt the assessment and provide a rest period or terminate the visit.

### Patient and Public Involvement

This study was designed with patient centricity as a core principle. Insights from thematic analysis of formative preprotocol need-gathering interviews with 6 patients with chronic pain informed the selection of multimodal data streams (narratives, drawings, and mobility). Patients will also be active participants in the formative evaluation and iterative design process in phase 2 of the study.

### Study Setting and Participants

The study will be conducted at up to 3 tertiary care academic interventional pain management practices in the United States. A centralized training program will be implemented for all research coordinators to ensure methodological fidelity. Key inclusion criteria include age of 18 years or above, a diagnosis of chronic pain (pain for >3 months), and the ability to provide written informed consent. Key exclusion criteria are conditions that could interfere with study procedures (eg, severe cognitive impairment), active litigation related to the pain disorder, or inability to speak and read English (due to the lack of validated translations for study instruments at this stage of prototyping).

### Sample Size

The study will enroll up to 80 participants. The sample size of 40 per phase is consistent with established benchmarks for qualitative saturation in human factors research [17]. This volume is sufficient to generate a diverse training dataset for exploratory AI models while allowing for the detection of usability failure modes. While this observational study is not powered for definitive hypothesis testing, a sample of 40 per phase allows for the estimation of the variance of the “contextual disconnect” rate to inform power calculations for future studies. Formal power calculations for clinical efficacy are not applicable due to the exploratory nature of this study.

### Study Procedures

The operational workflow and sequential flow of activities for both phase 1 (need and gap collection) and phase 2 (formative prototype collection) of the CONNECT study are systematically organized into distinct research blocks, as depicted in Figure 3. This structure ensures that multimodal data collection—encompassing subjective pain narratives, patient-guided drawings, and standardized physical mobility assessments—is executed in a standardized, reproducible manner across all investigational sites.

**Figure 3.**
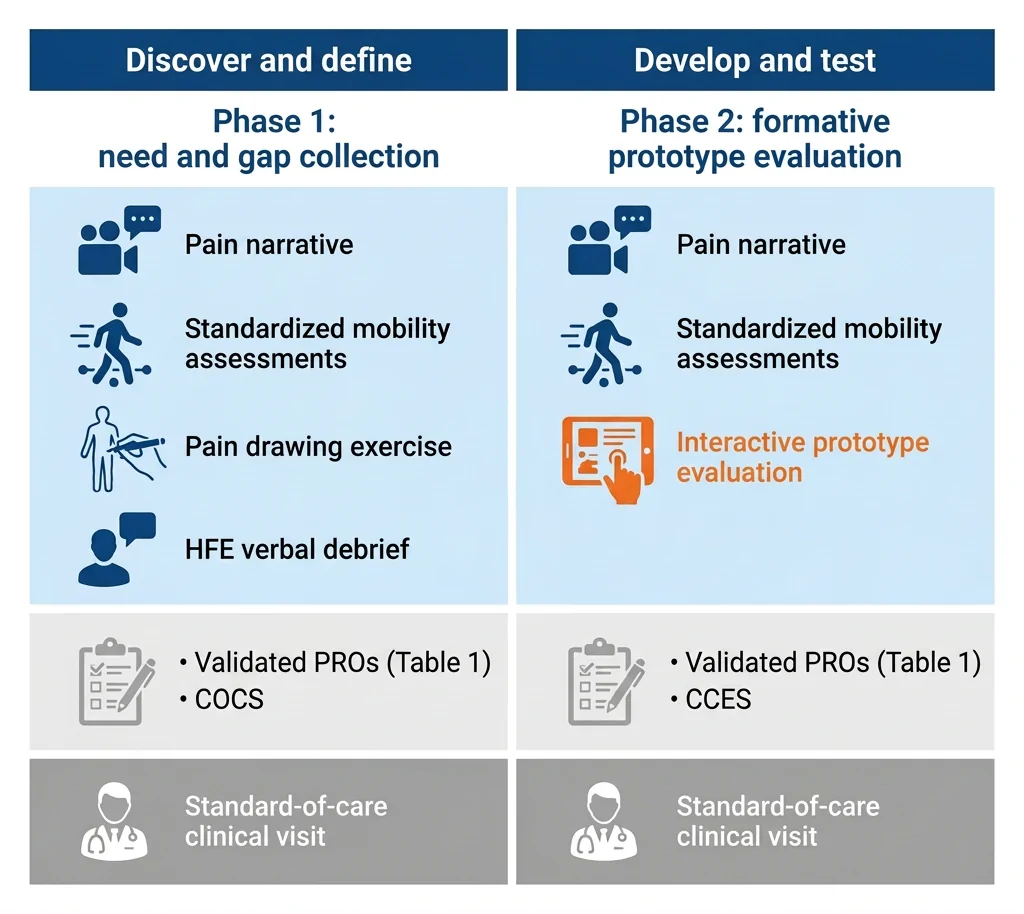
CONNECT (Collecting Communication Data to Enhance Patient-Physician Interaction) study procedures for phase 1 and phase 2. CCES: CONNECT Communication Efficacy Scale; COCS: CONNECT Open Communication Scale; HFE: human factors engineering; PRO: patient-reported outcomes.

#### Phase 1: Need and Gap Collection

##### Overview

Following informed consent, participants will complete a single study visit. To mitigate participant burden, the visit will be strictly capped at 90 minutes per the institutional review board (IRB)–approved protocol, and participants will receive a voucher in accordance with hospital policy. Patients will be monitored for pain exacerbation or distress and may pause or terminate the assessments at any time. It is important to note that this intensive, multimodal data collection is fundamentally required to generate rigorous scientific evidence and train the AI algorithms; it is not representative of the intended, streamlined real-world clinical workflow. The order of the 2 main components, a multimodal data collection session and a standard-of-care visit, will be randomized to mitigate procedural bias. The multimodal data collection session components are outlined in the following sections.

##### Function-Based Pain Narrative

This encompasses a video-recorded narrative initiated using a single open-ended verbal prompt to elicit the patient’s “pain story” followed by a fixed set of scripted follow-up questions to ensure data consistency across all participants.

##### Standardized Mobility Assessments

This encompasses video-recorded standardized tests, including a timed walk, sit-to-stand transitions, and an upper-body mobility sequence. To ensure safety, fall risk screening will be conducted prior to enrollment. Participants may use their standard assistive devices (eg, canes). Assessments will be immediately stopped if a participant requests to halt or exhibits signs of severe pain exacerbation or emotional distress.

##### Pain Drawing Exercise

This encompasses video-recorded free-form and structured, guided drawings of their pain with think-aloud verbal explanation.

##### Human Factors Engineering Verbal Debrief

This encompasses a video-recorded verbal interview and off-video human factors engineering (HFE) survey assessing the utility and perceived value of the multimodal data collection framework.

##### Validated Surveys

This encompasses validated patient-reported outcome (PRO) instruments (Table 1) and study-specific communication scales. Additionally, the Patient Activation Measure will be administered as a user-profiling covariate within the HFE assessment to contextualize usability feedback based on the patient’s self-management capability. Clinical site staff will be trained in best practices to help reduce potential bias from survey fatigue.

**Table 1.** Validated instruments for data collection.

Instrument name	Domain measured	References
NRS^a^	Pain intensity	[18]
MPS^b^	Pain severity and functional impact	[19]
PDI^c^	Pain-related disability	[20]
PROMIS-29^d^ Health Profile version 2.1	Multidimensional health (function, anxiety, etc)	[21]
PCS^e^	Pain-related thoughts and feelings	[22]
ODI^f^	Low back pain disability	[23]
CAT^g^	Physician communication skills	[24]
PHCPCS^h^	Quality of patient-provider communication	[25]
PAM-13^i^	Patient activation and self-management capability	[26]

a NRS: numeric rating scale.

b MPS: Mankoski Pain Scale.

c PDI: Pain Disability Index.

d PROMIS-29: Patient-Reported Outcomes Measurement Information System–29.

e PCS: Pain Catastrophizing Scale.

f ODI: Oswestry Disability Index.

g CAT: Communication Assessment Tool.

h PHCPCS: Patient-Health Care Provider Communication Scale.

i PAM-13: 13-item Patient Activation Measure.

### Phase 2: Human Factors Study and Prototype Development

This phase is a formative evaluation of up to 5 distinct software prototypes of the myStory tool. The order of multimodal data collection (as in phase 1) and an iterative prototype evaluation loop will be randomized, followed by a standard-of-care visit. The multimodal data collection session includes pain narratives and mobility assessments as in phase 1. During the prototype evaluation loop, participants will perform predefined tasks using each prototype while using a think-aloud protocol, followed by immediate usability and communication value assessments and PRO validated surveys.

No clinical intervention will be administered in this study. The myStory prototype is a nonprescriptive, general wellness tool used for utility and usability testing only. During this study, multimodal data will be collected strictly for research analysis and prototype development; treating clinicians will not have access to these data during standard-of-care visits. Ultimately, the intended future workflow involves patients using the tool before the visit and clinicians reviewing it within the visit.

### Outcomes

#### Overview

The study’s key primary and secondary outcomes are designed to assess the feasibility of data collection methods and quantify the clinical communication gap. The CONNECT Open Communication Scale (COCS) and CONNECT Communication Efficacy Scale (CCES) are novel, study-specific exploratory instruments (provided in full in Multimedia Appendix 1) designed to measure the primary feasibility end points. The conceptual framework and initial item wording for these scales were developed based on a thematic analysis of formative preprotocol patient interviews and consultation from subject matter experts in pain psychology and communication science. The cognitive debriefing interviews conducted during phase 1 will provide initial content validation for the final wording of these exploratory scales. The COCS (2 items) and CCES (5 items) are scored based on a 5-point Likert scale (range 1-5). Scale scores are calculated as the mean of the completed items, with no reverse-scored items. A minimum of 50% of the items must be completed to calculate a mean score; otherwise, the scale score will be treated as missing.

In addition to the primary acceptability scales, study feasibility will be evaluated using prespecified operational indicators. These include protocol completion rates, item-level missingness, technical failure rates, and the frequency of pauses or interruptions related to pain or distress.

For the purposes of this study, a key functional limitation (KFL) is defined as a significant life activity that a participant has stopped or has substantial difficulty performing due to pain, categorized using domains outlined in the Initiative on Methods, Measurement, and Pain Assessment in Clinical Trials recommendations [27]. On the basis of pilot data indicating that spoken narratives consistently yield the richest functional context, KFLs will be extracted primarily from the transcripts of the video-recorded pain stories. This extraction and coding will be performed by independent communication research partners using a dual coding and adjudication process. A contextual disconnect gap is defined as reporting both moderate or lower pain (NRS score of ≤6), a threshold consistent with published literature on pain severity [28], and the presence of at least one KFL. The threshold of an NRS score of 6 or less was selected as it represents mild to moderate pain intensity, a range in which severe functional limitations are frequently masked or underdocumented in standard clinical workflows. Exploratory sensitivity analyses assessing alternative NRS cutoffs and KFL severity levels may be conducted to further refine this metric.

The success criteria for each phase (summarized in Table 2) are outlined in the following sections.

**Table 2.** Primary and key secondary end points. The following sections provide details on the justification of the success criterion.

Study phase	End point type	Outcome measure	Success criterion	Purpose or what it validates
1	Primary	Mean score on the COCS^a^	>3.0	Feasibility of method: confirms that the open-ended narrative is a positive experience.
2	Primary	Mean score on the CCES^b^ for ≥1 prototype	>3.0	Feasibility of concept: confirms that a digital tool concept is perceived as effective as a communication utility.
1	Key secondary	Contextual disconnect rate (NRS^c^ ≤6 and ≥1 KFL^d^)	≥30%	Validation of clinical need: quantifies a meaningful gap in standard care.

a COCS: CONNECT Open Communication Scale.

b CCES: CONNECT Communication Efficacy Scale.

c NRS: numeric rating scale.

d KFL: key functional limitation.

#### Phase 1 Primary Outcome

The primary outcome for phase 1 is the mean score on the COCS, a 2-item scale assessing patient-perceived completeness and expressiveness of their narrative. The selected success criterion of a mean score greater than 3.0 is a standard feasibility benchmark for 5-point Likert scales and serves to indicate the feasibility of this communication method.

#### Phase 2 Primary Outcome

The primary outcome for phase 2 is the mean score on the CCES, a 5-item scale assessing the perceived effectiveness of the myStory elemental prototypes. The selected success criterion of a mean score greater than 3.0 is a standard feasibility benchmark for 5-point Likert scales and serves to indicate the feasibility of at least one prototype digital tool concept.

#### Key Secondary Outcome

The key secondary outcome is the prevalence of a contextual gap between patient-reported pain intensity (NRS ≤6) and self-reported KFLs (≥1). A contextual disconnect rate of 30% or higher (≥12/40 patients) is prespecified as clinically meaningful based on the Initiative on Methods, Measurement, and Pain Assessment in Clinical Trials recommendations, which designate a 30% prevalence to be meaningful, confirming the clinical need for a more holistic assessment tool [27,28].

#### Exploratory Outcomes

These include qualitative themes from the narrative analysis and pain drawing analysis; identification of any discordance between electronic medical record (EMR) documentation and pain narratives, rated using the EMR–Narrative Gap Assessment; identification of any discordance between patient drawings and EMR documentation; System Usability Scale scores, for which a score of 70 or higher is considered an above-average level of usability based on industry benchmarks [29]; and clinician-rated accuracy and utility of AI-generated narrative summaries, which will leverage automated detection of nonverbal affective cues grounded in the Facial Action Coding System [30-32].

### Data Analysis

#### Overview

The analytical approach for this study is designed to be transparent, descriptive, and appropriate for a human feasibility study. Given the exploratory nature of this feasibility study, formal inferential statistical tests for subgroup differences are not planned. An exploratory analysis of potential order effects will be conducted by stratifying key outcomes by randomization group. The analysis population will include all enrolled participants who complete the key procedures for their respective phase. If any data are suspected to be biased or inadequate (eg, based on research coordinator notes, identification of straight-lining, etc), a sensitivity analysis will be performed with those data removed, and the results of both the original analysis and the analysis with results removed will be reported. Quantitative analyses will focus on summarizing the primary and secondary end points, which include the COCS, CCES, and contextual disconnect rate, using standard descriptive statistics.

The exploratory end point analyses are structured to leverage the full multimodal dataset and detailed in the following sections. All AI-driven analyses are considered exploratory and hypothesis generating for future studies. Missing data will be documented and summarized descriptively.

#### Qualitative Exploratory Analysis

Qualitative data will be analyzed using a codebook-based thematic analysis approach. The initial thematic codebook was developed iteratively through a rigorous 6-step consensus process by 2 social scientists with expertise in thematic content analysis, mapping specific patient expressions to functional and emotional domains. A rigorous, systematic thematic analysis will be performed on all qualitative data (narratives, drawings, drawing commentaries, and open-ended interview responses) by 2 independent coders to ensure methodological fidelity following established guidelines for qualitative research. To establish the reliability of qualitative narrative transcript analysis, a randomly selected subset of narrative data (approximately 20%) will be dual coded, and interrater reliability will be formally assessed using the Cohen κ. A κ score of 0.70 or higher will be required to establish substantial agreement before the primary coder proceeds with full data coding [33,34]. Coding may be performed on text transcripts or directly from source media to preserve paralinguistic cues (tone and affect), with interrater reliability assessed on the coded output regardless of modality. In a secondary analysis, these themes will be mapped to the International Classification of Functioning, Disability, and Health, a World Health Organization global standard for describing health, to ensure that our findings are widely comparable [35,36].

For narrative analyses, qualitative themes, including the formal identification of patient-defined functional goals, will be extracted from narrative and pain drawing analysis. For drawing analyses, pain drawings will be analyzed using both thematic and anatomical coding using the same reliability procedures applied to narrative transcripts. For thematic analysis of AI-generated and human-coded narratives, the accuracy, clinical utility, and thematic concordance of AI-generated narrative summaries will be evaluated by comparing them with human-coded analyses. For thematic analysis of narratives and EMRs, the alignment between patient narratives and clinicians’ EMR documentation will be assessed using the EMR–Narrative Gap framework to characterize omissions, discrepancies, and qualitative mismatches.

#### Quantitative Exploratory Analyses

To leverage the multimodal data, several quantitative exploratory analyses are planned.

##### Pain Distribution Concordance

To quantify the “invisible pain” gap, the pain distribution concordance rate will be calculated as the percentage of participants with at least one major body region of pain indicated in their drawing but omitted from the corresponding EMR note.

##### Digital Biomarker (Kinematics)

To assess our digital biomarker hypothesis, objective kinematic variables (eg, gait asymmetry and sit-to-stand velocity) will be extracted from mobility videos using pose estimation models (eg, Google MediaPipe Solutions). Correlation coefficients will be calculated to explore the association between these kinematic markers and relevant PRO scores (eg, Patient-Reported Outcomes Measurement Information System–29 physical function domain). Additionally, an incongruence matrix will be generated for each participant, defined as a binary flag for any cases in which the NRS score is 6 or lower and the KFL count is one or more.

##### Usability Metric

To quantify the level of usability based on industry benchmarks, the System Usability Scale scores will be summarized using standard descriptive statistics.

### AI Model Development and Methodological Evaluation

#### Overview

Exploratory AI-assisted analyses will assess the methodological feasibility of applying machine learning techniques to extract features from multimodal data to support human interpretation. Commercial large language models (eg, GPT-5 [OpenAI] and Google Gemini class models accessed via secure enterprise APIs) will be used to generate narrative summaries and perform thematic content extraction on transcribed narratives. Established computer vision frameworks (eg, Google MediaPipe Solutions) will be used for pose estimation to identify movement patterns in mobility assessments. All identifiable audio and video data will be processed exclusively within a sponsor-controlled, HIPAA (Health Insurance Portability and Accountability Act)-compliant secure cloud environment; no identifiable media will be processed by unauthorized third-party systems. Model failures, hallucinations, and potential biases will be systematically documented during the human vs AI concordance evaluation. Specific model versions and configuration parameters will be documented in the final study report to ensure reproducibility.

The primary methodological evaluation of the AI will be a systematic comparison of the AI-generated themes against the human expert–coded analysis. In this study, we will define and use a multilayered framework for ground truth to ensure both patient-centered validity and clinical standardization.

#### First-Person (Phenomenological) Ground Truth

This consists of the raw, unfiltered, multimodal patient data (video narratives, drawings, and mobility assessments) captured during the in-clinic research visit. As the experience of pain is inherently subjective, the patient’s direct behavioral and verbal expression represents the primary, unmanipulated record of their lived experience, consistent with the International Association for the Study of Pain definition of pain [37] and US Food and Drug Administration guidance on PROs [38].

#### Third-Person (Clinical) Ground Truth

This is not a single data point but a composite of multiple expert analyses designed to capture different facets of clinical interpretation. It includes (1) structured thematic and KFL analysis (a rigorous, dual-coder thematic analysis of narrative transcripts performed by independent, experienced social scientists using a formal, predefined codebook to classify communication patterns and identify patient-reported KFLs), (2) unstructured clinical annotation (real-time think-aloud annotations from clinical investigators [eg, pain psychologist and interventional pain physician] as they review video data, capturing their unfiltered clinical impressions and reasoning), and (3) structured EMR–Narrative Gap assessment (a quantitative comparison performed by a pain psychologist using a study-specific instrument to rate the communication richness of the patient’s video narrative against their corresponding EMR note).

#### Ground Truth Integration and Reference Standard

This dual-layered model uses the first-person ground truth as the foundational experiential data and the composite third-person ground truth as the validated interpretive layer. Together, they form the reference standard against which all AI-generated summaries and myStory prototype outputs are evaluated. Concordance will be quantified using metrics such as the Jaccard index and Cohen κ to assess the degree of thematic overlap and reliability. These analyses are exploratory and will not influence primary or secondary end point evaluation.

### Data Collection, Management, and Security

Data will be collected using the sponsor’s HolisticCare Research Software on study-provided smart devices (iPhones and iPads; Apple Inc). All study data will be encrypted in transit (Transport Layer Security 1.2) and at rest (Advanced Encryption Standard–256) and stored in a secure, HIPAA-compliant environment controlled by the sponsor. A detailed cybersecurity management plan is in place to mitigate risks to data confidentiality, integrity, and availability. Access to identifiable data, particularly video recordings, is strictly limited to authorized study personnel via role-based access controls.

### Ethical Considerations

The study protocol (version C) was approved by the WCG central IRB (Puyallup, Washington, United States; approval 20254636 on November 24, 2025). WCG served as the central IRB for all participating sites. All participants will provide written informed consent prior to any study procedures. This feasibility study will not be registered in a public registry; this manuscript is intended to serve as the definitive public record of the prespecified study design. In the interest of full transparency, and to establish a permanent, citable public record of the prespecified design and analysis plan, this protocol is being published prior to the completion of enrollment.

### Dissemination Plan

The findings will be disseminated through presentations at scientific conferences and publications in peer-reviewed journals. Authorship will be determined in accordance with the International Committee of Medical Journal Editors criteria. As this is a noninterventional feasibility study, it was not formally registered in a public clinical trial registry.

## Results

Recruitment began in May 2026. As of September 2026, one site has been activated, 37 participants have been enrolled, and data collection is currently ongoing. Following the enrollment of the first participant, a minor procedural adjustment was implemented: the baseline data electronic case report form was moved to the end of the session sequence to prevent baseline survey questions from inadvertently biasing the patient’s unstructured pain narrative. No other procedures, end points, or planned analyses have been altered. First results are anticipated in late 2026. Formal analysis of the primary and secondary study end points has not yet commenced and is planned for late 2026 upon completion of full enrollment. However, an interim exploratory analysis of early phase 1 qualitative data (narratives and drawings) was conducted strictly to inform the functional parameters and interface design of the phase 2 myStory prototypes (in adherence to the human-centered, iterative design framework for this study). This exploratory analysis did not evaluate or influence any primary or secondary study end points.

## Discussion

### Anticipated Findings

This protocol outlines a comprehensive approach to capturing the holistic lived experience of patients with chronic pain. We anticipate that by systematically comparing multimodal data against standard NRS scores, we will identify a substantial contextual disconnect rate, demonstrating how capturing severe functional limitations provides critical context that complements the NRS. Furthermore, we expect that the phase 1 formative evaluation will confirm the technical feasibility and early patient acceptability of using an AI-driven, human-centered framework to capture these complex narratives.

### Comparison to Prior Work

The CONNECT study addresses a fundamental gap in chronic pain management by moving beyond simplistic pain scores to capture the holistic lived experience of patients with chronic pain. The strength of this protocol lies in its multimodal, human-centered design–driven methodology, which grounds technology development in a deep understanding of patient needs. By integrating video narratives, drawings, and objective mobility data, we aim to create a ground truth dataset that is far richer than what is typically available in clinical research. This work also aims to inform the creation of a practical clinical software tool. In HFE, this protocol represents an early formative evaluation. Its primary goal is to confirm user needs and assess whether this approach offers an acceptable alternative to the standard of care in the eyes of the patient (defined a priori as a mean Likert-scale score of >3.0). Establishing definitive clinical utility requires subsequent development phases outside this protocol, including iterative testing of physician and patient interfaces followed by longitudinal, real-world temporal exposure studies. By moving beyond text-only AI, our multimodal approach is better suited for the complexity of chronic pain. Crucially, by capturing patient-centered functional outcomes, this work provides the foundational data needed to demonstrate clinical and economic value in a value-based health care system [39]. The data from this study will be useful for both individualized care and system-level measurement [40].

Publishing this protocol prior to study completion is a commitment to scientific transparency and rigor. It establishes the prespecified end points, success criteria, and analytical methods, mitigating the risk of reporting bias.

### Strengths and Limitations

Limitations of this exploratory phase include the small sample size, single-visit design, reliance on proprietary tools, and exclusion of non–English-speaking participants due to a lack of translated instruments. The COCS and CCES are exploratory, study-specific instruments without established psychometric validity; findings based on these scales must be interpreted cautiously. Furthermore, the intensive nature of multimodal data collection (video narratives, drawings, surveys, and mobility testing) imposes a high participant burden. This may introduce selection bias as patients willing and able to complete these extensive procedures may have lower levels of pain, fatigue, or disability or higher baseline comfort with technology, which could affect the generalizability of the feasibility estimates. Additionally, this protocol does not assess clinical outcomes, which are reserved for subsequent phases of the CONNECT study. This current phase of research is designed to identify user needs and gaps in current health care delivery and provide a strong feasibility assessment and comparison of potential new approaches.

### Future Directions

The findings from the CONNECT study will not only quantify a critical communication paradox but will also provide the foundational evidence required to design and test a novel digital health software intervention in future, adequately powered validation studies. Ultimately, this research program aims to create tools that enhance therapeutic alignment, improve clinical efficiency, and restore the patient’s narrative to the center of their care.

## Supplementary material

10.2196/95222 Multimedia Appendix 1 Study-specific end point scales.

10.2196/95222 Checklist 1 SPIRIT checklist.

## Abbreviations

CCES: CONNECT Communication Efficacy Scale
COCS: CONNECT Open Communication Scale
CONNECT: Collecting Communication Data to Enhance Patient-Physician Interaction
EMR: electronic medical record
HFE: human factors engineering
HIPAA: Health Insurance Portability and Accountability Act
IRB: institutional review board
KFL: key functional limitation
NRS: numeric rating scale
PRO: patient-reported outcome
SPIRIT: Standard Protocol Items: Recommendations for Interventional Trials
