# Supplementary material for: A Human-Centered Framework to Capture the Holistic Pain Story in Chronic Pain: Protocol for a Multicenter Observational Feasibility Study
Source: JMIR Res Protoc. 2026 Oct 9;15:e95222. doi: 10.2196/95222 (PMC13651979; doi:10.2196/95222)
Supplement: Supplementary file 1 [file resprot-v15-e95222-s001.docx]

# Multimedia Appendix 2:

**Communication Assessment Instruments (COCs, CCES)**

**A. The CONNECT Open Communication Scale (COCS)**

- **Purpose:** To measure the patient-rated efficacy of the open-ended communication format used in Phase 1. This is the Primary Endpoint for Phase 1.
- ***Rationale*:** Designed to measure how effectively patients feel they can express their pain story using open-ended formats compared to traditional clinical visits.
- **Administration:** Administered to Phase 1 participants via the study tablet after the pain narrative and follow-up question tasks are complete.
- **Instrument:** Text:

**Instructions to Participant:**

"*Think about how you shared your pain story today using the open-ended format. Please rate how well this format helped you express your experience compared to a typical doctor visit."*

1. **Communication Completeness:** "How **complete** was your pain story today, compared to how you usually describe it during a typical doctor visit?"

*(Consider whether this format helped you include more of your experience, like emotions, body sensations, or daily impacts, than you usually get to share.)*

[1] Much less complete [2] A little less complete [3] About the same [4] A little more complete [5] Much more complete

2. **Expressive Facilitation:** "Were you able to express parts of your pain that you’ve had trouble explaining in past clinic visits?"

[1] Strongly Disagree [2] Disagree [3] Neutral [4] Agree [5] Strongly Agree

**B. The CONNECT Communication Efficacy Scale (CCES)**

- **Purpose:** To measure the patient-rated improvement in communication efficacy after interacting with the 'myStory' prototype. This is the Primary Endpoint for Phase 2.
- **Rationale:** Evaluates the perceived impact of the myStory prototype on communication clarity, emotional expression, and satisfaction.
- **Administration:** Administered to Phase 2 participants via the study tablet after the prototype usability testing is complete.
- **Instrument** Text:

**Instructions to Participant:**

"*Think about your experience using the 'myStory' tool today. Please rate how well it helped you communicate your pain story.*"

1. **Overall Communication:** "How **complete** was your pain story when using the tool, compared to how you usually explain it during a clinic visit?"

[1] Much less complete [2] A little less complete [3] About the same [4] A little more complete [5] Much more complete

2. **Expression of Detail:** "Did the tool help you express details that are usually hard to explain?"

[1] Strongly disagree [2] Disagree [3] Neutral [4] Agree [5] Strongly agree

3. **Confidence in Clinician Understanding:** "Did using the tool make you feel more confident that a clinician could understand your pain?"

[1] Strongly disagree [2] Disagree [3] Neutral [4] Agree [5] Strongly agree

4. **Ease of Expression:** "Using the tool helped me find words or ways to describe my pain more easily."

[1] Strongly disagree [2] Disagree [3] Neutral [4] Agree [5] Strongly agree

5. **Overall Satisfaction:** "Overall, how satisfied are you with using this tool to communicate your pain story?"

[1] Very dissatisfied [2] Dissatisfied [3] Neutral [4] Satisfied [5] Very satisfied
