# Supplementary material for: A Human-Centered Framework to Capture the Holistic Pain Story in Chronic Pain: Protocol for a Multicenter Observational Feasibility Study
Source: JMIR Res Protoc. 2026 Oct 9;15:e95222. doi: 10.2196/95222 (PMC13651979; doi:10.2196/95222)
Supplement: Supplementary file 2 [file resprot-v15-e95222-s002.docx]

# Multimedia Appendix 1:

**SPIRIT 2025 Checklist: Completed for the CONNECT Study (ABT-CIP-10586)**

| **Section / Topic** | **Item #** | **Location in CONNECT CIP & Page #** | **Checklist Item Content (As per CIP)** |
| --- | --- | --- | --- |
| **Administrative Information** |  |  |  |
| Title & Summary | 1a-b | Page 1, 72 | **Title:** CONNECT: COllectiNg commuNication data to Enhance patient-physiCian inTeraction.  **Design:** Multi-phase exploratory feasibility study.  **Population:** Chronic pain patients.  **Intervention:** N/A (observational and usability testing).  **Objective:** Assess feasibility of multimodal communication data capture.  **Trial Registration:** N/A, this publication serves as the public record.  **Sponsor:** Abbott.  **Data Sharing Statement:** De-identified data may be retained for future research; no formal plan for external sharing. |
| Protocol version | 2 | Page 1, 53 | Version C, IRB approved November 24, 2025. This version reflects updates made in response to IRB feedback on Version A, including clarification of endpoints and statistical methods, and updates to Rev B to shift from a single-site study to a multi-site study. |
| Roles and responsibilities | 3a-d | Sections 1.0, 10.0, App IX; Pages 1, 39-40, 70 | **Contributors:** The study protocol was designed by a multi-disciplinary team at the Sponsor (Abbott). Key contributors to the protocol's design include the PI (G. Yeganeh) and Co-I (K. Patel) who provided clinical oversight and expertise, and Dr. Maddie Gittleman, who provided expert psychological input on the design of participant-facing scripts and data collection instruments.  **AI Contribution:** Artificial intelligence technologies are a core component of the study's data analysis methodology. These include: (1) Natural Language Processing (NLP) pipelines, used to transcribe and extract thematic and functional features from patient narrative videos; and (2) Pose estimation models, used to extract quantitative kinematic variables (e.g., gait asymmetry) from the standardized mobility assessment videos. The Abbott technical team is responsible for the development and application of these technologies.  **Sponsor Role (Abbott):** Responsible for study design, conduct, management, data analysis, and has final authority on publication (CIP 14.0).  **Committees:** No formal Data Monitoring Committee (DMC) is utilized, which is appropriate for a low-risk feasibility study. |
| **Open Science** |  |  |  |
| Trial registration | 4 | Section 14.0, Page 46 | **N/A.** The study will not be registered. The stated intent is for this peer-reviewed protocol manuscript to serve as the definitive, citable, and public record of the study design and pre-specified endpoints. |
| Protocol & SAP | 5 | Main Body, Section 8.0, Page 33 | The full study protocol is detailed within this manuscript. The full statistical analysis plan (SAP) is described in Section 8.0 of the CIP (and this manuscript). |
| Data sharing | 6 | Section 11.3, Page 44 | **What will be shared:** Fully de-identified data derived from the study may be retained indefinitely for future internal research.  **How it will be shared:** A formal data sharing plan for making data available to external parties has not been established at the time of this protocol's publication. |
| Funding & COI | 7a-b | Section 10.3, Page 40 | **Funding:** The study is funded entirely by Abbott Laboratories.  **Conflicts of Interest:** Investigator Dr. Kiran Patel serves as an advisor/consultant for Abbott. Protocol authors are employees of Abbott. These will be formally declared in the manuscript. |
| Dissemination policy | 8 | Section 14.0, Page 46 | Results will be disseminated via peer-reviewed publications and conference presentations. Authorship will adhere to ICMJE guidelines. The Sponsor (Abbott) retains the right to review and approve all proposed publications. |
| **Introduction** |  |  |  |
| Background & Rationale | 9a-b | Section 1.0, Page 5 | Background details the failure of NRS to capture the lived experience of chronic pain. Rationale is to establish a framework for capturing multimodal "ground truth" data to inform better digital health tools. Comparator is the current standard of care (NRS-based assessment). |
| Objectives | 10 | Section 2.1, 4.1, Pages 6, 21 | To assess the feasibility of the data collection methods (Phase 1) and evaluate the usability and perceived communication value of a prototype digital tool (*myStory*) (Phase 2). |
| **Methods: Patient and Public Involvement, Trial Design** |  |  |  |
| Patient & Public Involvement | 11 | Section 1.0 (Rationale), Page 5 | The study design was directly informed by a thematic analysis of formative, pre-protocol interviews (N=6) with chronic pain patients. The Human-Centered Design (HCD) process places the patient at the center of the formative evaluation in Phase 2. |
| Trial design | 12 | Section 3.0, Page 8 | Multi-phase, multi-site, exploratory feasibility study with two mutually exclusive parallel cohorts (N=40 each). Randomization is used for the *order of assessments*, not for intervention allocation. |
| **Methods: Participants, Interventions, and Outcomes** |  |  |  |
| Trial Setting | 13 | Section 1.0, Page 1 | The study will be conducted at **up to three tertiary care academic interventional pain management practices in the United States.** To ensure consistency across sites, all study personnel will receive standardized training, and data collection procedures will be centrally harmonized. |
| Eligibility criteria | 14a | Sections 5.4 & 5.5, Pages 25-26 | Inclusion: Adults (≥18) with chronic pain (>3mo). Exclusion: Pregnancy, active litigation, non-English speaking, medical conditions that would interfere with assessments. |
| Intervention & Comparator | 15a-d | Section 2.2, Pages 6-7 | **N/A - Non-interventional.** The "intervention" is a usability test of a non-functional software prototype (*myStory*) in Phase 2 only. This is not a clinical intervention. The comparator is the patient's experience with standard clinical communication. No concomitant care is prohibited. |
| Outcomes | 16 | Sections 4.2-4.4, Pages 21-23 | **Primary (P1):** Mean score on the COCS.  **Primary (P2):** Mean score on the CCES.  **Secondary**: Prevalence of NRS/KFL contextual gap. Outcomes chosen to assess feasibility of communication methods and quantify the clinical communication gap. |
| Harms | 17 | Section 7.0, App V, Pages 30, 56 | Minimal risks anticipated (e.g., visual fatigue, discomfort during mobility tests). AEs related to study procedures will be monitored via passive surveillance by the Research Coordinator and reported. |
| Participant timeline | 18 | Section 5.9, Page 27 | Single study visit per participant. The timeline of assessments within the visit is detailed in flowcharts (Figures 2 & 5). |
| Sample size | 19 | Section 5.8, Page 27 | N=40 per phase. This is a feasibility study; a formal power calculation was not performed. The sample size is considered sufficient to detect usability issues and establish preliminary feasibility signals. |
| Recruitment | 20 | Section 5.2, 5.3, Pages 24-25 | Participants will be recruited from the clinical population at the study site by the site's research team. Stratified enrollment targets are in place to promote diversity. |
| **Methods: Assignment, Data Collection, Management, and Analysis** |  |  |  |
| Randomization & Blinding | 21-24 | Section 3.2, Pages 9-10 | **N/A for intervention.** Randomization is used to assign the *order of assessments* (e.g., storytelling before or after the clinic visit) to minimize procedural bias. It is unblinded. |
| Data collection & management | 25-26 | Sections 3.3, 6.0, 11.0, App IX | Data collection is performed by a trained Research Coordinator using standardized scripts and instruments on validated hardware/software (HCRS). Data management plan includes secure, encrypted cloud storage, role-based access, and a full cybersecurity plan (Appendix IX). |
| Statistical methods | 27a-d | Section 8.0, Pages 33-38 | Primary/secondary outcomes analyzed with descriptive statistics. Qualitative data analyzed via rigorous thematic analysis (dual coders, Kappa ≥ 0.70). An exploratory analysis of potential order effects will be conducted by stratifying results by randomization group. |
| **Methods: Monitoring** |  |  |  |
| Monitoring | 28-29 | Sections 10.6, 10.8, Page 42 | No formal DMC is planned, which is appropriate for a low-risk, non-interventional feasibility study. The Sponsor (Abbott) will perform study monitoring according to a pre-specified monitoring plan. |
| **Ethics** |  |  |  |
| Ethics approval & Amendments | 30-31 | Sections 12.1, 10.4, Pages 44, 40 | The study protocol (Version C) and ICF were reviewed and approved by **WCG Central Institutional Review Board** (IRB), a U.S.-based independent ethics committee. on November 24, 2025 (IRB# 20254636). All participating sites will operate under the authority of this central IRB. |
| Consent or assent | 32a-b | Sections 5.2, 12.1, Pages 24, 44 | Written informed consent obtained by a trained RC from all participants. The ICF explicitly details video recording, limits to de-identification, and data handling. No ancillary studies are planned. |
| Confidentiality | 33 | Sections 11.2, App IX, Pages 44, 70 | Participant data is coded with a unique ID. Identifiable video data is stored encrypted with strict role-based access controls. All data handling and security measures are detailed in the Cybersecurity Management Plan (Appendix IX). |
| Ancillary and post-trial care | 34 | Section 15.1, Page 46 | No direct benefit or post-trial care is planned. This is a single-visit, non-interventional study. |
